# The role of the midcingulate cortex in monitoring others' decisions

**DOI:** 10.3389/fnins.2013.00251

**Published:** 2013-12-20

**Authors:** Matthew A. J. Apps, Patricia L. Lockwood, Joshua H. Balsters

**Affiliations:** ^1^Nuffield Department of Clinical Neuroscience, University of Oxford, John Radcliffe HospitalOxford, UK; ^2^Department of Experimental Psychology, University of OxfordOxford, UK; ^3^Department of Psychology, Royal Holloway, University of LondonLondon, UK; ^4^Division of Psychology and Language Sciences, University College LondonLondon, UK; ^5^Neural Control of Movement Lab, Department of Health Sciences and Technology, ETH ZurichZurich, Switzerland; ^6^Trinity College Institute of Neuroscience, Trinity College DublinDublin, Ireland

**Keywords:** social reward, autism spectrum disorders (ASD), psychopathy, prediction error, midcingulate cortex, anterior cingulate cortex, social cognition, empathy

## Abstract

A plethora of research has implicated the cingulate cortex in the processing of social information (i.e., processing elicited by, about, and directed toward others) and reward-related information that guides decision-making. However, it is often overlooked that there is variability in the cytoarchitectonic properties *and* anatomical connections across the cingulate cortex, which is indicative of functional variability. Here we review evidence from lesion, single-unit recording and functional imaging studies. Taken together, these support the claim that the processing of information that has the greatest influence on social behavior can be localized to the gyral surface of the midcingulate cortex (MCC_g_). We propose that the MCC_g_ is engaged when predicting and monitoring the outcomes of decisions during social interactions. In particular, the MCC_g_ processes statistical information that tracks the extent to which the outcomes of decisions meet goals when interacting with others. We provide a novel framework for the computational mechanisms that underpin such social information processing in the MCC_g_. This framework provides testable hypotheses for the social deficits displayed in autism spectrum disorders and psychopathy.

Primates live in social environments that require individuals to understand the complex behavior of conspecifics. A plethora of research implicates the dorsal Anterior Cingulate Cortex (ACC) as playing a vital role in processing “social” information (i.e., processing elicited by, about, or directed toward others) (Amodio and Frith, 2006; Somerville et al., 2006; Rudebeck et al., 2008; Behrens et al., 2009; Apps et al., 2012; Hillman and Bilkey, 2012). Indeed, individuals with lesions to the ACC display social deficits so severe that they are said to have “acquired sociopathy” (Anderson et al., 1999). However, the ACC is also engaged by rewards (Doya, 2008), attention and salience (Davis et al., 2005), conflict, and during decision-making (Botvinick et al., 1999; Botvinick, 2007) which are inherently non-social processes. How can the same region be engaged by such a distinct set of processes? It is often overlooked that the area labeled as “ACC” by functional imaging research comprises multiple sub-regions, each with distinct cytoarchitecture and anatomical connections (Vogt et al., 1995; Palomero-Gallagher et al., 2008; Beckmann et al., 2009). Thus, some of the processes that have been reported to elicit an ACC response may in fact be localized to distinct sub-regions.

Here, we draw attention to anatomical tracer, neurophysiology, lesion and neuroimaging studies investigating the anatomical and functional properties of the dorsal ACC. Taken together this research highlights one sub-region which processes information about the outcomes of others' decisions and about the decisions made by others during social interactions. This region in fact lies on the gyral surface of the midcingulate cortex (MCC_g_) and not in the anatomically defined ACC. We contend that whilst the sulcal (MCC_s_) and gyral (MCC_g_) regions of the MCC can be differentiated in terms of processing first-person and social information respectively, the two areas process similar information about rewards that guide decision-making. By drawing parallels between the role of the MCC_s_ in processing first-person rewards, and that of the MCC_g_ in processing rewards in social contexts, we provide a new framework for investigating the contribution of the MCC to social decision-making.

## Anatomy of the cingulate cortex

**Figure 1:**
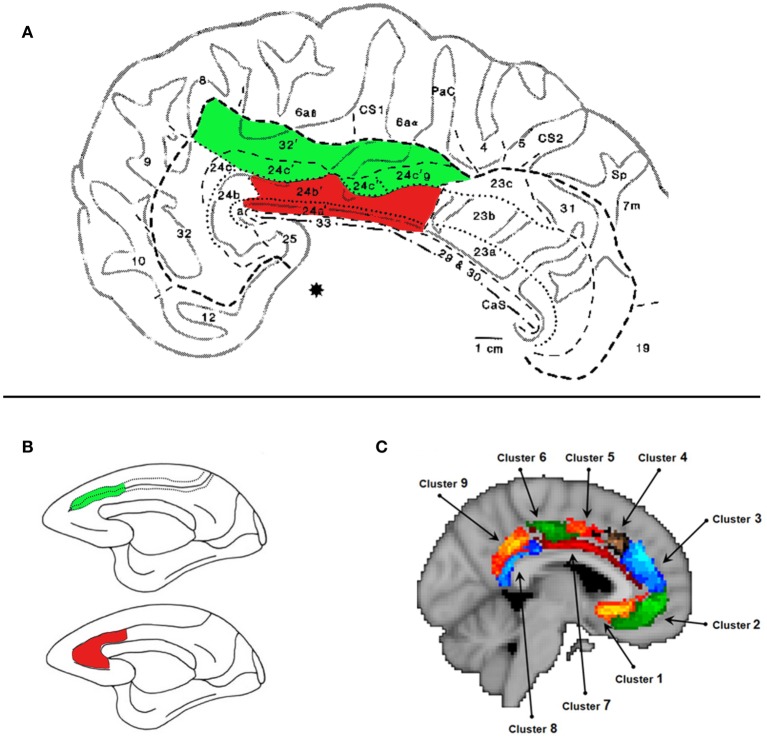
**The Midcingulate Cortex (MCC)**. **(A)** Cytoarchitecture of the MCC taken from Vogt et al. (1995). The areas shaded in green lie in the MCC_s_. The areas shaded in red lie on the MCC_g_. We argue that this area is engaged when processing information about others' decisions. Specifically we argue that areas 24a' and 24b', which lie on gyral surface of the cingulate cortex, extending on average 22 mm posterior to and 30 mm anterior to the anterior commisure denoted by (*). **(B)** Lesion site of the MCC_g_ and ACC_g_ (red) and the MCC_s_ and the ACC_s_ (green) from Rudebeck et al. (2006). The lesions that affected the gyrus caused disruptions to social behavior and disrupted the processing of social stimuli. **(C)** Subdivisions of the MCC and ACC according resting-state connectivity (Beckmann et al., 2009). Cluster 7 shown in dark red corresponds, broadly, to the MCC_g_.

The cingulate cortex consists of four zones: retrosplenial, posterior (PCC), mid (MCC), and anterior (ACC) (Vogt et al., 1987, 1995; Palomero-Gallagher et al., 2008). Often the MCC is labeled as “dorsal” ACC and the actual ACC as “rostral” ACC. Unfortunately, the use of ACC as a “catch-all” terminology, has led many to inaccurately discuss the functional properties of an MCC result in relation to the functional and anatomical properties of the ACC. The ACC and MCC can be further subdivided by their cytoarchitecture (Palomero-Gallagher et al., 2008). In both the MCC and ACC there are differences in cytoarchitecture between the sulcus and the gyrus (see Figure 1A), indicative of distinct functional properties. Notably in this article we are discussing only regions within the cingulate cortex and not the region lying at the borders of the paracingulate sulcus and the superior frontal gyrus (“paracingulate cortex”) that is well known for its role in processing social information.

Each cytoarchitectonic region has a different connectional fingerprint (Vogt and Pandya, 1987; Vogt et al., 1987; Devinsky et al., 1995; Margulies et al., 2007; Beckmann et al., 2009; Torta and Cauda, 2011). The MCC_g_ shows a connectional profile that suggests involvement in processing information about others. This region has been shown to have strong connections with posterior portions of the superior temporal sulcus (pSTS) (Pandya et al., 1981; Seltzer and Pandya, 1989), temporal poles (TPs) (Markowitsch et al., 1985; Barbas et al., 1999) and paracingulate cortex (Vogt and Pandya, 1987; Petrides and Pandya, 2006). These areas have been consistently linked to processing information about others' mental states and intentions (Frith and Frith, 2003; Ramnani and Miall, 2004; Amodio and Frith, 2006; Hampton et al., 2008). There is minimal overlap between these connections and those of other portions of the ACC and MCC to the TPs, the pSTS and paracingulate cortex. Furthermore, the tracer studies listed above suggest that connections between the MCC_g_ and these areas may be stronger than the connections from other ACC and MCC sub-regions. This profile leads us to propose that the MCC_g_ is the sub-region of the cingulate cortex that plays the most significant role in social behavior.

Interestingly, the MCC_g_ has connections which overlap with the MCC_s_ to areas that are engaged during reward-based decision-making. Both areas project to medial and lateral portions of the orbitofrontal cortex (Morecraft et al., 1992; Morecraft and Van Hoesen, 1998) and to the nucleus accumbens (Kunishio and Haber, 1994; Haber et al., 1995). Anterior portions of both MCC sub-regions also receive dopaminergic input from the ventral tegmental area (VTA) (Hollerman and Schultz, 1998; Schultz, 1998; Williams and Goldman-Rakic, 1998). The connections of both the MCC_g_ and MCC_s_ to areas engaged when processing rewards (Schultz, 2006; Rushworth and Behrens, 2008) are indicative of a shared sensitivity to information that guides decision-making. Thus, we suggest that the MCC_g_ plays an important role in processing information about the rewards others will receive and the decisions that lead to others' rewarding outcomes.

## The MCCg and social information processing

Is there functional evidence for a role of the MCC_g_ in processing reward-related information that guides decisions during social interactions? Chang et al. (2013) recorded from single-neurons during a task where monkeys received rewards or when they observed another monkey receiving reinforcement. They found a class of neurons lying on the gyral surface putatively in the MCC (although without histology it is not possible to localize accurately) that showed a change in spike-frequency when the monkeys observed another receiving the reward. The same neurons did not respond on trials when the monkeys received a reward themselves. Only a small proportion of neurons in the MCC_s_ showed this same profile. This response profile highlights the MCC_g_ as signaling information related to outcomes experienced by others (i.e., it contains a class of neurons that respond exclusively to others' reward receipt). Whilst only one study, this supports our claim that the MCC*_g_* processes information about rewards that others will receive.

Evidence from lesion studies also supports the notion that the MCC_g_ processes social information. Lesions to the gyrus of the MCC and ACC of macaques have been shown to reduce the execution of social behaviors, such as the time spent in proximity with others and vocalizations, and also the processing of social stimuli (Hadland et al., 2003; Rudebeck et al., 2006). Unoperated monkeys or those with lesions to the MCC_s_ or to the OFC, show delays in responding to a food item in the presence of social stimuli. Monkeys with lesions to the MCC_g_ (Figure 1) show a reduced delay, suggesting a reduction in the value assigned to the social information (Rudebeck et al., 2006).

**Figure 2:**
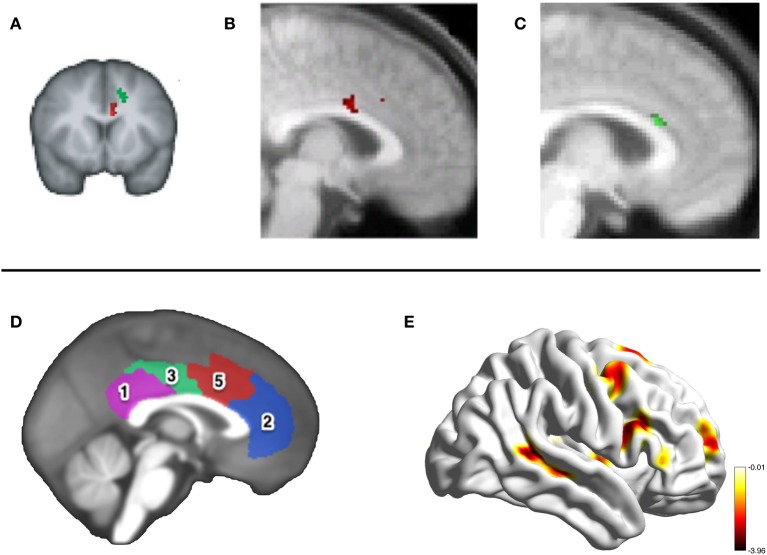
**Neuroimaging the MCC**. The top panel shows activity in the same portion of the MCC_g_ in three fMRI studies investigating reward processing during social interactions. **(A)** Activity in the MCC_g_ (the cluster in red, MNI coordinate: −6, 12, 26) correlating with the volatility of advice given by a social confederate on a reward-based decision-making task, taken from Behrens et al. (2008). Activity in this cluster correlated with individual differences in the influence that the advice had on the subjects' own decision-making. **(B)** Activity in the MCC_g_ [taken from Apps et al. (2013)] signaling a prediction error when the outcome of another's decision was unexpectedly positive (coordinate: 0, 8, 28), but not to the expected or unexpected outcomes of a computer's responses. **(C)** Activity shown in the MCC_g_ (coordinate: 4, 22, 20) correlating with the anticipated net-value (benefit-cost) of a reward to be received by another person, but not rewards that will be received one's self [taken from Apps and Ramnani (under review)]. The bottom panel shows the results of resting-state connectivity analysis in Autism Spectrum Disorders by Balsters et al. (in prep). Connectivity between the MCC, cluster 5 shown in red **(D)**, and the pSTS **(E)** was reduced in ASD compared to control participants.

A small number of neuroimaging studies in humans have tested the claim that it is the MCC_g_ and not the MCC_s_ which processes information about others' decision-making. In Behrens et al. (2008) participants learned the probability of receiving a rewarding outcome from two options associated with different reward levels. On each trial participants received advice from a confederate about which option to choose. To maximize financial return subjects had to track how volatile the environment was (how rapidly the better option was shifting between the two) and also the volatility of the confederate advice. Whilst MCC_s_ activity covaried with the environmental volatility, activity in the MCC_g_ covaried with the volatility of the advice at the time of every trial outcome (Figure 2A).

Apps et al. (2013) examined activity when participants monitored the decisions and outcomes of a confederate and a computer, when the outcomes were sometimes unexpectedly either positive or negative. They examined activity at the time of a cue that revealed the outcome of the trial to the subject before it was revealed to the confederate or computer. Whilst the MCC_s_ signaled when the outcome of either the computer or confederate's response was unexpectedly positive, the MCC_g_ signaled the same information but only when the choice was made by another person and not by the computer (Figure 2B). Unpublished data from Apps and Ramnani (under review), also found that the MCC_g_ signaled the net-value of rewards others will receive (benefit-cost) and not the net-value of one's own rewarding outcomes. These findings support the claim that the MCC*_g_* is engaged when processing information about the rewards others receive (Figure 2C).

## The MCC_s_, decision-making and response-outcome monitoring

Whilst there has been considerable theoretical discussion of the functional properties of the MCC (or “dorsal ACC”), this literature largely ignores the contribution of this region to social cognition and is based on studies that find activation that lies predominantly, or exclusively, in the MCC_s_. As a result, there is a an absence of a theory of MCC_g_ function. However, it is notable that the studies discussed in the previous section are consistent with a claim that the MCC_g_ processes similar information to the MCC_s_. Here, we discuss a theoretical account of MCC_s_ function, in order to draw parallels with the MCC_g_ in the next section.

Recent theoretical accounts suggest that the MCC_s_ is engaged when predictions are made about the outcomes of decisions and when the outcomes of decisions are monitored (Alexander and Brown, 2011; Silvetti et al., 2013). When outcomes are discrepant from those that were predicted, neurons in the MCC_s_ signal prediction errors (PE), equating to the surprise evoked by the outcome (Matsumoto et al., 2007; Holroyd and Coles, 2008; Quilodran et al., 2008; Jocham et al., 2009; Kennerley et al., 2011; Nee et al., 2011). Furthermore, it has been argued that such a response-outcome functional property allows the region to play a role in monitoring the extent to which behaviors are meeting higher order needs or goals (Behrens et al., 2007; Botvinick, 2012; Holroyd and Yeung, 2012; Kolling et al., 2012). That is, the MCC tracks response-outcome contingencies within the context of how actions are meeting temporally abstract goals. Although there is not scope to discuss studies in detail here, there is evidence that MCC prediction and outcome processing is modulated by the extent to which behaviors are meeting contextually driven goals (Behrens et al., 2007; Rushworth and Behrens, 2008; Kolling et al., 2012).

It has been suggested that information processing in the MCC conforms to the principles of hierarchical reinforcement learning theory (HRL). In HRL, learning is not simply between stimulus-response and outcome [as in classic reinforcement learning (RL)], but learning occurs in a hierarchical framework where multiple actions (or sub-goals) must be performed and monitored in order to reach the higher-order goal (e.g., stimulus-response-response-response-outcome learning) (Botvinick, 2012). As such, each performed action is aimed at meeting a sub-goal that does not lead to a rewarding outcome on its own, but the performance of each action is crucial in order to achieve the higher order goal of the rewarding outcome. In HRL PE signals drive learning and occur when an outcome is unexpected as in RL. There are a considerable number of neurophysiological and neuroimaging studies have shown that neurons in the MCC_s_ signal when the outcomes of decisions are unexpected (Matsumoto et al., 2007; Holroyd and Coles, 2008; Quilodran et al., 2008; Jocham et al., 2009; Kennerley et al., 2011; Nee et al., 2011). However, unlike in standard RL, in HRL PEs occur when actions fail to achieve sub-goals. These are sometimes referred to as pseudo-prediction errors (PPE) as they are not directly linked to the receipt of a rewarding outcome. Ribas-Fernandes et al. (2011) showed that the MCC_s_ signal occurs when a PPE would be processed and not at the time when a classic PE would be signaled. This suggests that the PE signals in the MCC_s_ may operate to track the extent to which an action is meeting an organism's goals by signaling the surprise at the time of the outcome of a decision. These surprise signals may take the form of PPEs as proposed in HRL.

## The MCC_g_ : predictions and errors during social interactions

We argue that the MCC_g_ processes similar information to the MCC_s_ but does so during social interactions [i.e., information is processed in an “other” reference frame (Hunt and Behrens, 2011)]. That is, the MCC_g_ signals predictions and monitors outcomes during social interactions when the outcome will be received by another. We suggest that social behavior can be organized into a HRL framework, whereby a subject's own goal of how to interact with another acts as a higher-order policy. The actions of others (or one's own actions impacting upon another) will therefore serve as sub-goals to that policy. The outcome of each action (or sub-goal) will be monitored during a social exchange, in relation to the prior predictions instantiated by the higher-order goal. Thus, we suggest the MCC_g_ will be engaged when processing the value of each action during a social exchange. In addition, it will be involved in processing information about whether actions or choices meet current, overarching goals in a social environment. When a sub-goal is not met, a “social” prediction error (SPE) will signal the discrepancy between the predicted and actual consequences of the choice, whether self or other, updating the agent's own policy. Simply put, the MCC_g_ will signal predictions and monitor the outcomes of each action when interacting with another. However, the nature of the predictions will be influenced by the context within which each action and outcome are being processed. Thus, the context of a social interaction will influence the manner in which the MCC_g_ codes information about others' rewarding outcomes.

For this theoretical account to hold true,the MCC*_g_* must be sensitive to rewards that others receive, MCC_g_ activity must be related to higher level statistical properties of others' behavior (e.g., volatility) and it must signal prediction errors when the outcomes of others' choices are unexpected. These three properties were demonstrated in studies outlined above, where we highlighted that the MCC_g_ contained neurons that responded when another receives a reward (Chang et al., 2013), MCC_g_ activity tracked the volatility of another's choices (Behrens et al., 2008) and also this area signalled when the outcome of another's decision was unexpected (Apps et al., 2013). Furthermore, this account would also allow for considerable flexibility and individual differences in how reward-related information is processed in different social contexts, and therefore the extent to which MCC*_g_* influences behavior.

## The MCC_g_ and disorders of social cognition

What predictions can be made for behavioral consequences of MCC_g_ damage? We suggest that disruptions to the MCC_g_ will have two main effects: first, this account would be a multi-faceted impact on motivation for engagement in social interactions may decline as decreased sensitivity to others' rewards will diminish the influence of such outcomes on the higher-order goals of an agent. Furthermore, when presented with the possibility of interacting with another, the motivation for attending to sub-goals will not be maintained and agents may become apathetic toward social engagement. In addition, even when engaged in a social interaction, a failure to maintain motivation for attending to sub-goals would result in unsustained social interaction. Second, we contend that MCC_g_ dysfunction may cause a failure in individuals to update the value of a policy when an unexpected outcome of a sub-goal fails to evoke a SPE. As a result, an agent may become insensitive to an outcome of a sub-goal that reduces the value of a reward another will receive (i.e., a reduction in empathy), or to the outcomes of their own actions that reduce the value of a rewarding outcome for another (e.g., a failure to maintain prosocial behaviors).

The first prediction fits with existing theories of social deficits displayed in Autism Spectrum Disorders (ASD) (Dawson et al., 2005; Chevallier et al., 2012). Social Motivation Theory (Chevallier et al., 2012) proposes that individuals with ASD are unable to form stimulus-reward contingencies for social stimuli, resulting in reduced social attention and engagement. Chevallier et al. (2012) focused on an orbitofrontal-striatal-amygdala circuit; we propose that the MCC_g_ may play a key role in ASD. Previous studies have shown disturbed cytoarchitecture specifically in the MCC_g_ in individuals with ASD(Simms et al., 2009). Similarly, Delmonte et al. (2013) showed hyperconnectivity between the caudate and MCC_g_ in children with ASD, the strength of which was negatively correlated with neural responses to social rewards (Delmonte et al., 2012). Unpublished data by Balsters et al. (in prep) suggests a reduction in connectivity between the MCC and the pSTS, an area that is engaged when processing others' mental states, in individuals with ASD (see Figure 2).

A meta-analysis of fMRI studies examining social processing in ASD compared to controls (Di Martino et al., 2009). They showed consistent group differences in anterior and posterior regions of the cingulate cortex in the processing of social stimuli, but not in the MCCg for either the social or non-social tasks. However, our theoretical perspective would suggest that differences in MCCg function in ASD will only be observed when processing others' decisions or outcomes during social interactions. To date, studies examining social processing in ASD and those reviewed in the meta-analysis, have largely focused on the perception of social stimuli and not required subjects to interact with another and monitor decision-outcome contingencies. Future research should therefore test the tenets of our theory specifically when subject are engaged in a social interaction.

The second prediction above matches behavioral deficits seen in individuals with psychopathy, who are suggested to be insensitive to rewards that others will receive, leading to increased competitive behaviors (Mokros et al., 2008; Koenigs et al., 2010; Curry et al., 2011). Similarly, individuals with psychopathy have been shown to display a reduced error related negativity, measured using Electroencephalography, when observing other's outcomes during a social interaction (Brazil et al., 2011). This signal is putatively sourced in the MCC. Recent studies also indicate that gray matter volume and activity in the MCC_g_ correlate with psychopathic and callous traits (De Brito et al., 2009; Anderson and Kiehl, 2012; Cope et al., 2012; Lockwood et al., 2013). Thus, whilst only preliminary evidence, these studies highlight the putative role that differences in MCC*_g_* function may have to psychopathy and psychopathic traits and particularly to the choices they make when interacting others.

## Summary

Based on anatomical connectivity, neurophysiology and neuroimaging evidence, we suggest that the region of the cingulate cortex that plays the most important role in social cognition and social behavior lies in the MCC_g_. Our model highlights this region as playing an important role in predicting and monitoring the outcomes one's own and others' decisions when the outcomes will be experienced by another. Future research should examine the extent to which the MCC_g_ is engaged when monitoring the outcomes of others' decisions and how deficits in MCC_g_ function lead to deficits in using social information to guide one's behavior.

### Conflict of interest statement

The authors declare that the research was conducted in the absence of any commercial or financial relationships that could be construed as a potential conflict of interest.
